# Anesthetic Management of a Patient with Harlequin Ichthyosis

**DOI:** 10.1155/2021/9953320

**Published:** 2021-07-26

**Authors:** Klint J. Smart, Catherine A. Gruffi, Tara M. Doherty

**Affiliations:** Department of Anesthesiology, Westchester Medical Center, 100 Woods Road, Valhalla 10595, NY, USA

## Abstract

Harlequin ichthyosis is a severe and often fatal form of congenital ichthyosis caused by defective lipid transport which results in a dysfunctional skin barrier. Patients who survive the neonatal period are predisposed to skin infections, sepsis, impaired thermoregulation, and dehydration. The unique skin characteristics can present significant anesthetic challenges. We highlight the relevant anesthetic considerations in a 3-year-old presenting for syndactyly release of the right second and fourth digits. We describe the steps to ensure protection of the fragile skin barrier during establishment of intravenous access and airway management, therefore providing guidance for care of this vulnerable patient population.

## 1. Introduction

Harlequin ichthyosis (HI) is the most severe form of autosomal recessive ichthyosis and is caused by a mutation in the ABCA12 gene which is involved in lipid transport to the skin surface [1, 2]. Neonatal presentation is distinctive and distressing, as the infant is encased in thick layers of skin separated by deep diamond-shaped fissures covering the entire body [3]. These plaques place patients at increased risk for dehydration, thermoregulatory dysfunction, sepsis, and respiratory failure in the immediate neonatal period [3, 4]. Treatment with oral retinoids and topical emollients can encourage plaque shedding via skin turnover but result in excessively tight skin and diffuse erythroderma [3]. Other sequelae of HI include ectropion, eclabion, frequent skin infections, heat and cold intolerance, and digital contractures [3, 5]. High-calorie diet supplementation and frequent dilute hypochlorite bleach baths with chronic emollient application are common challenges for caretakers [6]. It is imperative that anesthesia providers are well educated about this unparalleled skin condition when these patients are scheduled for surgery in an effort to minimize their risk. Illustrating the anesthetic techniques executed in order to achieve the least harm can be beneficial to future providers when a patient with HI is encountered.

This manuscript adheres to the applicable Enhancing the Quality and Transparency of Health Research (EQUATOR) guideline as well as the CARE (CAse REports) guidelines.

## 2. Case Description

A 3-year-old girl with a past medical history of harlequin ichthyosis and syndactyly was scheduled for syndactyly release of the right second and fourth digits as well as an excisional biopsy of a left shoulder mass. She was born via cesarean section at 34 weeks and required escharotomies of the hands and feet bilaterally while in the neonatal intensive care unit. Significant history included multiple admissions for methicillin-susceptible *Staphylococcus aureus* bacteremia and urinary tract infections as a result of her skin condition. She also had a history of difficult intravenous access that previously required traumatic intraosseous lines and a peripherally inserted central catheter during prior hospitalization. Physical examination showed diffusely erythematous skin along with areas of abnormal keratinization and large areas of desquamation all over the patient's body. She had hallmark ectropion eyelids that were unable to close fully and limited mouth closure. There were no visible or palpable veins observed in the patient's extremities.

**Figure 1:**
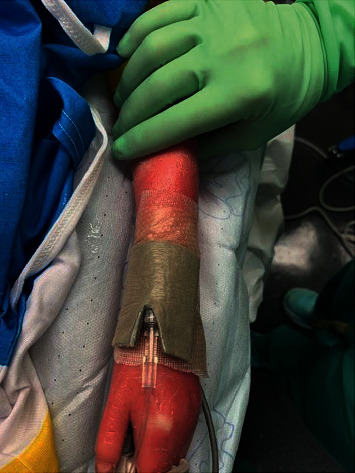
ADAPTIC® nonadherent dressing underneath the catheter reinforced with Mepilex® Border Ag antimicrobial dressing.

Prior to surgery, the patient was premedicated with 0.5 mg/kg of oral midazolam with good effect and proceeded to the operating room with parental presence. Once in the operating room, a finger clip pulse oximeter was placed to avoid direct adhesive effect of the standard wrap pulse oximeter. The blood pressure cuff was placed over the patient's clothing on the lower extremity in order to avoid direct pressure on the skin. Preoperatively, her parents had been counseled about possible needle-based EKG monitors and they elected to forgo EKG monitoring. Resta™ moisturizing ointment was carefully placed on the patient's facemask, and general anesthesia was induced with incremental inhaled sevoflurane. The patient's eyes were then liberally lubricated with both 0.5% erythromycin ophthalmic ointment as well as GenTeal® eye ointment. Under ultrasound guidance, a 22-gauge intravenous (IV) catheter was placed in the patient's left forearm on the first attempt. Initially, nonadherent ADAPTIC® dressing was placed underneath the IV line connection. Next, Mepilex® antimicrobial foam dressing was placed over the nonadherent ADAPTIC® mesh (Figure 1) and secured with a 3M™ Coban™ self-adherent wrap.

**Figure 2:**
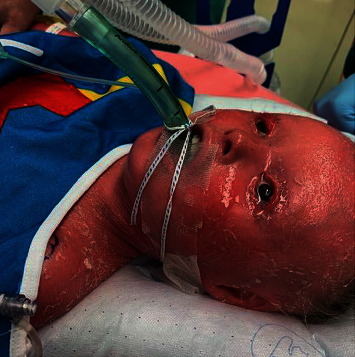
Laryngeal mask airway (LMA) secured with an umbilical tape and ADAPTIC® dressing.

After the IV catheter was carefully secured, 50 mg of propofol was administered and a size 2.5 Ambu® laryngeal mask airway (LMA) was placed with ease. The LMA was secured with a Ethicon® cotton umbilical tape in a circumferential manner around the head. ADAPTIC®, a nonadherent dressing, was used for the insertion site of the IV to prevent skin maceration. This dressing was also placed between her skin and the umbilical tape (Figure 2). General anesthesia was maintained with sevoflurane, multimodal analgesics, and local infiltration at the operative sites. Prophylactic antiemetics included ondansetron and metoclopramide. Careful temperature monitoring was pursued, and normothermia was maintained with an underbody Bair Hugger™. After an uneventful case, the LMA was removed awake, the patient was transferred to the postoperative anesthesia care unit, and she was discharged home successfully on the day of surgery. She was seen at a follow-up visit 1 week later and was noted to have healed well without any ill sequelae from the procedure or the anesthetic.

## 3. Discussion

At birth, neonates with HI have the unique appearance of truncal-shaped plates of skin with deep fissuring, bilateral ectropion, and eclabium. The neonates that survive into infancy develop severe ichthyosiform erythroderma (50%) and palmoplantar keratoderma (25%)^2^. As a result of their compromised skin barrier, these patients are at risk for impaired thermoregulation, sepsis, and dehydration^3^. Their unique skin features can present significant challenges to the anesthesiologist. Fragile, hyperkeratotic skin confounds routine IV placement, and chronic emollient use interferes with securing lines and tubes and routine monitors.

The ADAPTIC® dressing was ideally suited for this case as it is formulated with a cellulose acetate mesh and petroleum which was ideal for her fragile skin. As was the Mepilex® Border Ag antimicrobial dressing which was placed over the nonadherent dressing. The adhesive in these dressings have a low potential for skin irritation and trauma while providing a barrier for infectious pathogens. Both dressings were easily removed without evidence of any damage to the skin. An LMA was favored over an ETT for this case but either could have been used and secured with intraoral sutures or an umbilical tape. Purposeful avoidance of dexamethasone for nausea prophylaxis was chosen because of possible impaired wound healing.

In summary, HI is associated with characteristic skin features that make multiple aspects of an anesthetic challenging. We found insufficient literature describing details of anesthetic procedures in patients with HI and wanted to share our experience. This case report demonstrates the successful steps utilized to place and secure the customary lines and tubes associated with a routine anesthetic in order to prevent harm and improve patient satisfaction.

## Data Availability

The data for this case report includes the clinical details from the anesthetic encounter as discussed in the manuscript as well as the information obtained by the cited references. There are no restrictions to data access.

## Abbreviations

ASA: American Society of Anesthesiologist
ECG: Electrocardiogram
EQUATOR: Enhancing the Quality and Transparency of Health Research
HI: Harlequin ichthyosis
IV: Intravenous
LMA: Laryngeal mask airway
ABCA12: Adenosine triphosphate binding cassette A12.
